# Interparental Conflict and Early Adolescent Depressive Symptoms: Parent-Child Triangulation as the Mediator and Grandparent Support as the Moderator

**DOI:** 10.1007/s10964-023-01923-2

**Published:** 2023-12-13

**Authors:** Meiping Wang, Shan Sun, Xiaojie Liu, Yang Yang, Chunyu Liu, Aodi Huang, Siwei Liu

**Affiliations:** 1 Department of Psychology, Shandong Normal University, Jinan, China; 2 Department of Student Affairs Management, Jining College, Qufu, China; 3 Department of Student Affairs Management, Yantai University, Yantai, China; 4 Department of Human Ecology, University of California, Davis, Davis, CA USA

**Keywords:** Interparental conflict, Parent-child triangulation, Grandparent support, Early adolescent, Depressive symptoms

## Abstract

A notable ambiguity persists concerning whether distinct forms of parent-child triangulation (unstable coercive coalition, stable coalition, detouring-attacking, detouring supportive, parentification) might mediate the association between interparental conflict and early adolescent depressive symptoms similarly within the context of Chinese Confucianism. Filling this research gap, this study aimed to examine the mediating role of the five dimensions of parent-child triangulation in the association between interparental conflict and early adolescent depressive symptoms, as well as the moderating effect of grandparent support on this mediating pathway. Data were drawn from a sample of 761 Chinese adolescents (*M*_age_ = 12.82 ± 0.47, 49.1% girls). Structural equation model analyses indicated that unstable coercive coalition, stable coalition, and detouring-attacking behaviors partially mediated the association between interparental conflict and adolescent depressive symptoms, while detouring-supportive behaviors and parentification did not demonstrate such mediating effects. Unlike Western societies, a negative correlation was observed between interparental conflict and parentification in the context of China. Grandparent support mitigated the adverse effects of both interparental conflict and the unstable coercive coalition on early adolescent depressive symptoms.

## Introduction

Early adolescence is a crucial period for investigating susceptibility to depression, as it is characterized by a notable surge in both the prevalence and intensity of depressive symptoms (Frey et al., 2020). Depressive symptoms among early adolescents are related to a variety of psychosocial and behavioral maladjustments, such as substance abuse, psychological imbalances, and suicide, and usually persist into adulthood (Gijzen et al., 2021; McLeod et al., 2016). It is essential to examine the influencing factors and the underlying mechanisms of early adolescent depressive symptoms. According to the family systems theory (Minuchin, 1985), the family is conceptualized as a structured entity consisting of multiple subsystems, including marital relationships, parent-child relationships, and grandparent-child relationships, all of which jointly influence child and adolescent development. To fully understand the etiology of adolescent depressive symptoms, it is necessary to simultaneously examine the functional dynamics of these subsystems within the context of the family. Guided by the family systems theory, the present study aimed to better illuminate the influence of interparental conflict on early adolescent depressive symptoms, with parent-child triangulation as a potential mediator and grandparent support as a moderator.

### Interparental Conflict and Depressive Symptoms

It has been well established that interparental conflict, as a pivotal metric of marital relationships, contributes to internalizing and externalizing problems in children and adolescents (Qi, 2021; Ran et al., 2021). Both cross-sectional and longitudinal studies have consistently supported the association between interparental conflict and adolescents’ adjustment problems, including depressive symptoms (Asanjarani et al., 2022; Luo et al., 2023).

The existing literature suggests several possible mechanisms underlying the linkage between interparental conflict and child adjustment, such as the structural family systems perspective (Minuchin, 1974), the cognitive-contextual model (Grych & Fincham, 1990), and the emotional security hypothesis (Davies & Cummings, 1994). The structural family systems perspective postulates that the diffused boundaries between parents and children may lead to the formation of cross-generation coalitions that inappropriately involve children in the parental subsystem, thus posing developmental risks for children (Minuchin, 1974). The cognitive-contextual model asserts that children’s appraisals of interparental conflict play a crucial role in their adjustment. When children feel responsible for their parents’ discord or perceive themselves as threatened, they may develop a strong desire to intervene in these disputes, potentially leading to adverse consequences for the children (Grych & Fincham, 1990). The emotional security hypothesis suggests that when interparental conflicts undermine children’s sense of emotional security, children may try to mitigate these conflicts, thereby putting them in a distressing position (Davies & Cummings, 1994). Parent-child triangulation has been considered to be a central theme in these theoretical models (Kerig & Swanson, 2010). As such, parent-child triangulation has attracted increasing attention and has been considered one important family process underlying the linkage between interparental conflict and child adjustment.

### Parent-child Triangulation as a Mediator

The concept of triangulation was developed by Bowen (1978), who proposed in his family systems theory that it is an extremely common but disordered way for two people in a family (generally parents) to manage their conflicts and tensions by pestering a third party (usually a child) (Wang et al., 2017). To maintain the balance of the family system, children are actively or passively involved in conflicts, forming a “parent-child triangulation”, a particular form of parent-child relationship that includes three forms: cross-generational coalitions, scapegoating, and parentification (Kerr & Bowen, 1988). In cross-generational coalition, a child forms a coalition with one of the parents against the other in a parental conflict (Minuchin, 1974). Cross-generational coalition can be either unstable or stable. The former denotes situations where children align with either parent at different times, while the latter signifies a long-term and stable alliance between a child and one parent. Scapegoating, a way of parents avoiding or resolving their conflict by diverting their attention onto the child, involves detouring-attacking and detouring-supportive (Kerig & Swanson, 2010). The former refers to parents forming a coalition against the child with problematic behaviors, such as addiction to games, violence, or learning problems, in which the child is rejected or perceived as a problem (Kerig, 2016). The latter refers to parents concealing their conflict by paying too much attention to their child’s well-being, in which the child is cared for or considered special, delicate, or needy (Kerig & Swanson, 2010). Parentification means the roles of parents and children are reversed as children try to resolve parental conflicts by providing caregiving and support to their parents (Kerr & Bowen, 1988).

Empirical research has demonstrated a significant association between interparental conflict and the emergence of parent-child triangulation, with heightened interparental conflict fostering triangulation behaviors (Camisasca et al., 2019). Parent-child triangulation has also been observed as an impact on child psychosocial adjustment. When a child embroiled in parent-child triangulation attempts to mitigate tensions and preserve balance during episodes of interparental conflict, their likelihood of experiencing externalizing and internalizing problems increases (Grych et al., 2004). Therefore, parent-child triangulation has been hypothesized to be a mediator between interparental conflict and child adjustment. Several studies have examined the mediating role of parent-child triangulation, and have indeed provided supportive evidence (Grych et al., 2004; Franck & Buehler, 2007).

However, some other work has implied that the association between parent-child triangulation and child development may vary across the dimensions of triangulation and cultural contexts. Contrary to the compelling evidence on the adverse effect of cross-generational coalition and scapegoating on child adjustment (Coe et al., 2020; Wang et al., 2017), there have been mixed findings on the association between parentification and child adjustment. Some studies conducted in Western societies (Goldner et al., 2022; Pakenham & Cox, 2012) indicated that increased engagement in parentification was linked with poorer adjustment. However, other studies suggested that enrollment in parentification could promote adjustment, especially within the Chinese context (Kerig, 2005; Wang et al., 2017). The disparities in these findings may be due to the different operational mechanisms underlying the various dimensions of parent-child triangulation (Yang, 2011), as well as differences in cultural values between the East and the West. The cross-generational coalition is characterized by ‘loyalty’, leading children to grapple with loyalty conflicts regardless of their alignment with mother or father (Peterson & Zill, 1986). The scapegoating dimension operates through a ‘guilt’ mechanism, with children perceiving themselves as undesirable and attributing themselves as the primary culprits in interparental conflicts and disharmony (Minuchin, 1974). Parentification, in contrary, operates through a “responsibility” mechanism, as children in this role prioritize maintaining family harmony over their own self-care (Wells & Jones, 2000). Influenced by Confucian culture, family harmony is regarded as a shared duty among family members (Liu, 2003). Adolescent parentification is encouraged as a positive behavior that instills a sense of value and fosters positive social adjustment in teenagers (Wang & Wang, 2014). Conversely, Western cultures place a high value on individual rights, independence, and personal will (Hong, 2007). Parentification implies sacrificing personal aspirations for the family’s well-being, which may not be beneficial to individual future development.

Most existing studies on parent-child triangulation have overwhelmingly focused on one or two dimensions (coalition, scapegoating, or parentification) or on an aggregate score of triangulations, which reflects the extent to which children feel caught in the middle of parental conflict. Few studies have explicitly examined the potential distinctions between unstable and stable coalitions, or between detouring-attacking and detouring-supportive behaviors. In particular, no study so far has assessed the five forms of parent-child triangulation simultaneously. Although it is reasonable to posit that parent-child triangulation as a whole can mediate the association between interparental conflict and depressive symptoms in early adolescence, it is unclear whether its distinct forms (unstable coercive coalition, stable coalition, detouring-attacking, detouring supportive, and parentification) play similar roles. It is particularly important to examine these relations within the context of China, where family processes are profoundly influenced by Confucianism, which emphasizes family harmony, members’ responsibility, obligation, and sacrifices to the family (Bond, 2010).

### Grandparent Support as a Moderator

Extant evidence has shown that social support is strongly correlated with depressive symptoms (Scardera et al., 2020). According to the Stress-Buffering Model (Rueger et al., 2016), social support serves as a protective factor for individuals in the face of stressful events. During adolescence, social support can buffer the harmful effects of adverse environment on psychological adjustment (Duru et al., 2019). Within the Chinese context, one important type of social support during adolescence is grandparent support, which refers to the support grandchildren receive from their grandparents. Influenced by traditional culture, it is common for Chinese grandparents to be involved in parenting (Luo et al., 2020). Grandparents often act as a replacement in the absence of parent company (Van Heerden & Wild, 2018), providing children love and support (He & Ye, 2014). Meanwhile, grandparents may influence the social and emotional development of children and adolescents by regulating the interactions between family members (Zhang et al., 2022).

Empirical research has shed light on the role of grandparent support in the psychological adjustment of adolescents. For example, grandparent support could promote adolescent prosocial behavior (Profe & Wild, 2017) and psychological well-being (Huang, 2010). Grandmothers’ involvement in parenting played a protective role in the development of children exposed to harsh parenting by their mothers (Barnett et al., 2010). A recent study with Chinese adolescents as participants indicated that grandparent support could mitigate the adverse impact of a lack of coparental cooperation on adolescents’ depression (Zhang et al., 2022). In research focusing on depression, although previous studies have examined the role of social support, most have focused on parental and peer support. Overall, grandparent support has got limited attention within the domain of adolescent depressive literature, even in the Chinese context. Few studies have examined the moderating effect of social support on the association between interparental conflict and adolescent depressive symptoms. To our knowledge, only one study investigated the moderating role of sibling warmth (sibling support) on the association between exposure to interparental conflict and adolescents’ depression (Tucker et al., 2013).

According to the family systems theory, the impact of both interparent conflict and parent-child triangulation on adolescents will likely depend on grandparent support, an index of grandparent-child relationships. Considering the documented protective effect of grandparent support, it is reasonable to expect that grandparent support can directly mitigate the detrimental consequences of interparental conflict on adolescents’ depressive symptoms. Grandparent support may also protect adolescents from involving in these unfavorable family processes (interparent conflict and parent-child triangulation), and thus decrease adolescents’ risk of depressive symptoms. It is also possible that adolescents with higher grandparent support possess a higher sense of security and attempt to resolve conflicts between parents, thereby putting them at heightened risk for depressive symptoms. The present study aims to explore whether and how grandparent support moderates the influence of interparental conflict on early adolescent depressive symptoms directly and indirectly.

## Current Study

**Fig. 1:**
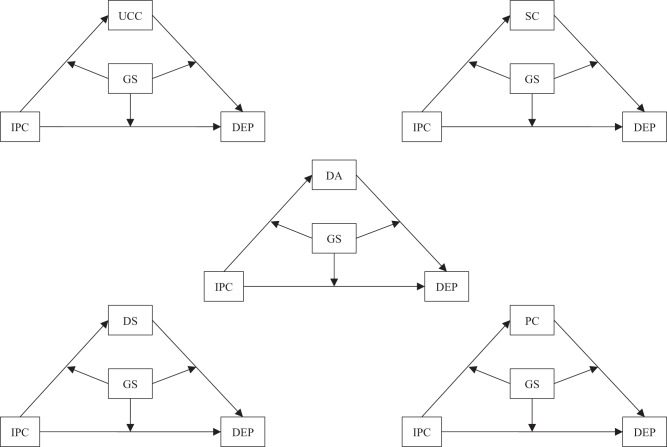
The moderated mediation model. UCC Unstable coercive coalition, SC Stable coalition, DA Detouring-attacking, PC Parentification, DS Detouring-supportive, IPC Interparental conflict, GS Grandparent support, DEP depressive symptoms (the following is the same)

While it was reasonable to hypothesize that parent-child triangulation as a whole could mediate the association between interparental conflict and depressive symptoms in early adolescence, it remained unclear whether its distinct forms might exhibit comparable mediating effects, particularly within the context of Chinese Confucianism. There was also a lack of comprehensive examination of the marital relationship, parent-child relationship, and grandparent-grandchild relationship in the existing literature. This study had two primary objectives. Firstly, this study explored whether the five dimensions of parent-child triangulation mediated the relation between interparental conflict and adolescent depressive symptoms differently, and compared these findings with prior research conducted in Western cultural contexts. Secondly, this study investigated the moderating role of grandparents in the aforementioned mediating pathways. It was hypothesized that parent-child triangulation would mediate the association between interparental conflict and adolescent depressive symptoms, and the mediation pathway would be moderated by grandparent support (see Fig. 1). However, no hypotheses were formulated regarding potential variations in the moderated mediation among specific forms of triangulation, primarily due to limited relevant evidence.

## Methods

### Participants

The sample size required for this study was estimated in advance using G*Power 3.1.9.2 software (Faul et al., 2009). A minimum sample size of 395 was estimated with a small effect size of *f*^*2*^ = 0.02 and a statistical test power of 0.8 (α = 0.05). In this study, the initial sample size was 820 subjects, and after excluding single-parent families (n = 34), reconstituted families (n = 16), and others (e.g., living in foster homes with relatives, n = 9), the final participants consisted of 761 (49.1% girls) adolescents who lived with their parents. The majority resided in a two-parent family (n = 652) and the remainder reported living in an extended family (n = 109). They were recruited from one elementary school (n = 252) and one junior high school (n = 509) in Qingdao, a coastal city on the east of China. Among them, 33.1% (n = 252, *M*_*age*_ = 11.83 ± 0.44, 122 girls) were in Grade 6, 32.2% (n = 245, *M*_*age*_ = 12.78 ± 0.47, 116 girls) were in Grade 7, and 34.7% (n = 264, *M*_*age*_ = 13.71 ± 0.50, 134 girls) were in Grade 8. Approximately 39.9% of adolescents have no siblings. A smaller percentage of parents (17.0% of mothers, 19.5% of fathers) had an education level higher than vocational school (e.g., college or graduate degree). One-third of parents (36.2% of mothers, 37.9% of fathers) had a high-school education background. 36.8% of mothers and 32.6% of fathers did not go beyond junior high school. A large proportion of the adolescents lived in a two-parent family (85.7%), while the others lived in an extended family (14.3%). Most adolescents were born in urban areas (81.7%), while the others were born in rural areas (18.3%). The median monthly family income was between $ 1025.

### Procedure

Adolescents were recruited by convenience sampling. Prior to data collection, the research gained approval from the ethics committee of Shandong Normal University. School principals were informed about the purpose, content, and duration of the investigation. Approval was also obtained from mothers or fathers during parent-teacher meetings, and from adolescents themselves at the investigation site. Nearly all parents and adolescents willingly participated, with a response rate of 98% for each grade. Well-trained graduate students in psychology carried out all data collection. During school time, they distributed a series of questionnaires and provided standardized instructions for completing the questionnaires. Participants were asked to complete the questionnaires independently and quietly in their classrooms, which took about 30 minutes. During this period, the participants’ school teachers were not allowed to be present. Small gifts such as notebooks and pencils were given to adolescents for their participation. Data confidentiality was rigorously maintained by ensuring that adolescents’ data would be used exclusively for scientific research and would not be shared with any other individual without their explicit consent.

### Measures

#### Interparental conflict

The Conflict Properties of the Children’s Perception of Interparental Conflict Scale developed by Chi and Xin (2003) was utilized as a measure of interparental conflict. This scale assessed the frequency, intensity, and resolution of interparental conflict and consisted of 19 items (e.g., “My parents have almost never quarreled.”) rated on a 4-point Likert scale (1 = totally agree, 2 = agree to an extent, 3 = disagree to an extent, 4 = totally disagree). Items were averaged with high scores indicating high levels of interparent conflict, that is, more frequent, intense, and poorly resolved conflict. The scale is widely used in Chinese adolescent populations and has been validated for its reliability and validity (Xiong et al., 2022). Cronbach’s α was 0.92 in this study.

#### Parent-child triangulation

Parent-child triangulation was assessed by a revised Family Triangulation Inventory, originally developed by Zhang (2000). This scale consisted of 23 items rated on a 3-point Likert scale (1 = totally disagree, 2 = agree to extent, 3 = totally agree), and include five dimensions: (i) unstable coercive coalition (4 items, e.g., “When my parents quarrel, I sometimes stand by my mother’s side and sometimes by my father’s side.”), (ii) stable coalition (4 items, e.g., “When my parents quarrel, I only speak for one of them.”), (iii) detouring-attacking (5 items, e.g., “When my parents quarrel, they will get angry with me.”), (iv) detouring-supportive (4 items, e.g., “When my parents quarrel, I find that I will be taken care of more than usual.”), and (v) parentification (6 items, e.g., “When my parents quarrel, I often calm them down.”). Confirmatory factor analysis (CFA) indicated that the revised Family Triangulation Inventory showed a good fit to the data (*χ*^2^/*df* = 2.00, CFI = 0.94, TLI = 0.93, SRMR = 0.05, RMSEA = 0.04). This scale has been widely used in studies with Chinese adolescents. Responses were averaged for each dimension, with higher scores representing more involvement in parent-child triangulation. In this study, Cronbach’s α for the five subscales were 0.70, 0.68, 0.78, 0.68 and 0.80, respectively.

#### Grandparent support

Grandparent support was measured using the Grandparent Support subscale of the Social Support Scale (Yan & Zheng, 2006). The scale is consisted of four items (e.g., “I can discuss my problems with my grandparents”) rated on a 7-point Likert scale (from 1 = extremely disagree to 7 = extremely disagree). This scale has been widely used in studies with Chinese adolescents (Kong et al., 2023; Ban et al., 2022). Adolescents self-report their perceived support from grandparents, with higher scores indicating greater grandparent support. Confirmatory factor analysis (CFA) indicated that the grandparent support subscale of the Social Support Scale fit the data well (*χ*^2^/*df* = 3.20, CFI = 0.97, TLI = 0.96, SRMR = 0.03, RMSEA = 0.06). For this study, its Cronbach’s α was 0.83.

#### Depressive symptoms

Adolescents reported their depressive symptoms on the Center for Epidemiologic Studies Depression Scale (CES-D) (Radloff, 1977). This scale is widely used by Chinese adolescents and has been validated for its reliability and validity (Chen et al., 2009). The CES-D consisted of 20 items (e.g., “I felt fearful.”) tapping adolescents’ feelings during the past week using a four-point scale (1 = occasionally or none, 2 = sometimes, 3 = half of the time, 4 = most or all-time), with higher scores indicating more severe symptoms of depression. The Cronbach’s α for CES-D was 0.89 in this study.

### Covariates

The following variables were included as covariates in the model, gender, grade, and urban/rurality.

### Data Analysis

First, all continuous variables were standardized before analysis to minimize multicollinearity. Descriptive statistics and bivariate Pearson correlations for the variables were calculated. Second, a series of structural mediation models were constructed to investigate whether the different dimensions of parent-child triangulation mediate the association between interparental conflict and depressive symptoms. Third, a series of moderated mediation models were built to explore whether grandparent support moderates the three paths of the mediation models. A simple slope test and the Johnson–Neyman (J-N) method were utilized to further reveal the possible moderating effect (Hayes, 2017). Significance tests and confidence interval (CI) estimates were performed by 5000 bootstrap samples. To control Type I error, *p*-values were adjusted using the Benjamini and Hochberg (B-H) procedure (Benjamini & Hochberg, 1995). In this study, SPSS 26.0, Mplus8.0, and PROCESS 4.0 were used for data analysis.

### Missing Data

Analyses showed that there was no missing data on the key variables, and the percentage of missing data for both adolescents’ gender and location (urban/rural) variables was less than 5%. The Littie’s missing completely at random (MCAR) test for both variables was not statistically significant (*χ*^2^ (2) = 0.29, *p* = 0.87), indicating that the missing data for both variables were completely at random. Given the small proportion of missingness and the type of missingness. Pairwise deletion (correlation analysis) and listwise deletion (mediation analysis and moderated mediation analysis) were used in subsequent analyses.

## Results

### Common Method Bias

To control the possible common method deviations resulted from self-report questionnaires, the measures were arranged separately, and anonymous survey and reverse scoring in some items were used. Harman’s single factor analysis was utilized to test common method deviations. The results indicated that 14 factors’ eigenvalues were greater than 1. The first factor explained 18.9 % of the total variance, which was less than the critical criterion of 40%, suggesting that no serious common method bias emerged in the study.

### Descriptive Statistics and Correlation Analysis

Means, standard deviations, and correlation coefficients for the variables are shown in Table 1. Interparental conflict was positively related to unstable coercive coalition, stable coalition, detouring-attacking, and depressive symptoms, and negatively correlated with detouring-supportive and parentification. Moreover, unstable coercive coalition, stable coalition, and detouring-attacking were positively linked with depressive symptoms, while detouring-supportive, parentification, and grandparent support were negatively associated with depressive symptoms. Being a girl, higher grade, and coming from a rural area were related to more depressive symptoms; thus, gender, grade, and urban/rurality were controlled as covariates in the following analyses. As parental education levels and family structure were not significantly associated with adolescents’ depressive symptoms, they were not considered in this study.

**Table 1:** Descriptive statistics and bivariate correlations of variables

Variable	1	2	3	4	5	6	7	8	9	10	11
1.Gender	–										
2.Grade	0.02	–									
3.U-R	–0.01	0.01	–								
4.IPC	–0.02	0.07	0.10^**^	–							
5.UCC	–0.04	–0.02	0.06	0.21^**^	–						
6.SC	–0.05	–0.03	0.07^*^	0.22^**^	0.17^**^	–					
7.DA	–0.07	0.05	0.05	0.43^**^	0.30^**^	0.34^**^	–				
8.DS	–0.07^*^	–0.10^**^	0.02	–0.08^*^	0.27^**^	0.15^**^	–0.01	–			
9.PC	–0.09^*^	–0.17^**^	–0.01	–0.30^**^	0.18^**^	0.02	–0.16^**^	0.33^**^	–		
10.GS	–0.01	–0.07^*^	–0.03	–0.30^**^	0.08^*^	0.01	–0.13^**^	0.25^**^	0.29^**^	–	
11.DP	0.08^*^	0.10^**^	0.09^*^	0.47^**^	0.17^**^	0.18^**^	0.37^**^	–0.09^*^	–0.22^**^	–0.24^**^	–
*M*	–	–	–	1.84	1.95	1.29	1.42	1.67	2.27	5.22	1.61
*SD*	–	–	–	0.57	0.57	0.40	0.44	0.51	0.54	1.34	0.52

“Gender” was a dummy variable, 0 = male, 1 = female; “U-R means urban/rurality”

*UCC* Unstable coercive coalition, *SC* Stable coalition, *DA* Detouring-attacking, *PC* Parentification, *DS* Detouring-supportive, *IPC* Interparental conflict, *GS* Grandparent support, *DEP* depressive symptoms

*p < 0.05; **p < 0.01

### Mediation Analysis

**Fig. 2:**
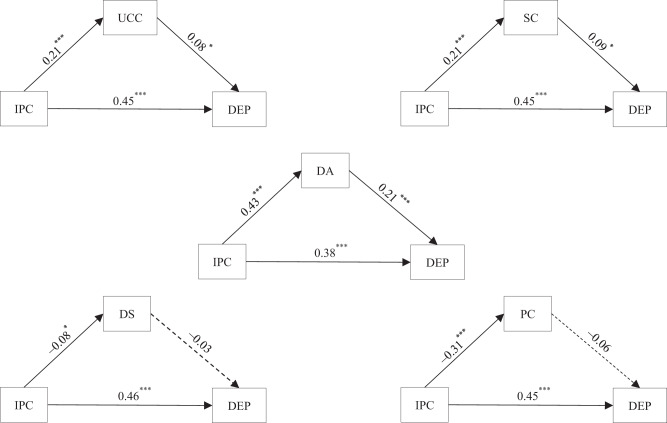
Testing the mediating effect of parent-child triangulation on the association between interparental conflict and early adolescent depressive symptoms. The numbers were standardized regression coefficients. For the sake of brevity, the control variables (gender, grade and urban/rurality) were not shown in the figure (the following is the same). UCC Unstable coercive coalition, SC Stable coalition, DA Detouring-attacking, PC Parentification, DS Detouring-supportive, IPC Interparental conflict, GS Grandparent support, DEP depressive symptoms. *p < 0.05, ***p < 0.001

After controlling for covariates, a positive effect of interparental conflict on depressive symptoms was found in the absence of parent-child triangulation (*b* = 0.47, *SE* = 0.03, *t* = 14.47, *p* < 0.001). Next, a series of mediation models were constructed with parent-child triangulation as the mediating variable (see Fig. 2). All models were found to fit the data well (*χ*^2^/*df* ≤ 2.49, CFI ≥ 0.97, TLI ≥ 0.93, SRMR ≤ 0.02, RMSEA ≤ 0.05). The direct effects of interparental conflict on adolescent depressive symptoms remained significant in all models (see Fig. 2). Moreover, unstable coercive coalition (proportion 3.62% of mediating effect = 0.02, 95% CI = [0.003, 0.04]), stable coalition (proportion 4.05% mediating effect = 0.02, 95% CI = [0.005, 0.04]) and detouring-attacking (proportion 18.98% mediating effect = 0.09, 95% CI = [0.06, 0.13]) partially mediated the relation between interparental conflict and depressive symptoms, while detouring-supportive and parentification did not.

### Moderated Mediation Analysis

Three moderated mediation models were constructed with unstable coercive coalition, stable coalition, and detouring-attacking as the respective mediators and grandparent support as the moderator. All of the models fit the data well (*χ*^2^/*df* ≤ 2.58, CFI ≥ 0.97, TLI ≥ 0.90, SRMR ≤ 0.02, RMSEA ≤ 0.05).

**Fig. 3:**
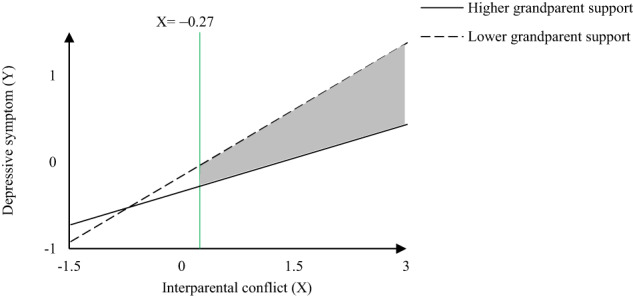
The effect of interparental conflict on the depressive symptoms, moderated by grandparent support with Johnson–Neyman confidence intervals indicating significant confidence regions

A significant interaction between interparental conflict and grandparent support on depressive symptoms was found in each model (see Tables 2–4). A simple slope test showed that the predictive effect of interparental conflict on depressive symptoms was tempered for adolescents with higher grandparent support (*B*_*high*_ = 0.27, *SE* = 0.05, *t* = 5.52, *p* < 0.001, 95% CI = [0.17, 0.36]; *B*_*low*_ = 0.51, *SE* = 0.04, *t* = 12.12, *p* < 0.001, 95% CI = [0.43, 0.59]). The Johnson–Neyman analyses further indicated that the buffering effect of grandparent support was significant when the interparental conflict score was more than –0.27 (*B*_*JN*_ = –0.07, *SE* = 0.04, *t* = 1.96, *p* = 0.05, 95% CI = [–0.14, 0.00]) (region depicted by a gray background in Fig. 3).

**Table 2:** The moderated mediation effect of unstable coercive coalition and grandparent support on the association between interparental conflict and early adolescent depressive symptoms

Variable	Equation1 (Dependent variable: UCC)				Equation2 (Dependent variable: DEP)			
	*b*	*t*	*p*	*p(i)*	*b*	*t*	*p*	*p(i)*
IPC(X)	0.27	7.10	**0.001**	0.003	0.39	11.28	**0.001**	0.003
UCC(M)					0.11	3.42	**0.001**	0.003
GS(U)	0.15	3.95	**0.001**	0.003	–0.09	–2.72	**0.007**	0.025
X*U	0.07	2.14	0.033	0.025	–0.12	–4.14	**0.001**	0.003
M*U					0.09	3.12	**0.002**	0.018
*R*^*2*^	0.08				0.28			
*F*	9.09^***^				32.46^***^			

The control variables (gender, grade and urban/rurality) were displayed in the model. *P* is the original *p*-value, *p(i)* is the critical value of significance level corrected by a B-H procedure, and the result is significant if *p* ≤ *p(i)*, significant results after B-H correction are highlighted by bold face, same below

*UCC* Unstable coercive coalition, *SC* Stable coalition, *DA* Detouring-attacking, *PC* Parentification, *DS* Detouring-supportive, *IPC* Interparental conflict, *GS* Grandparent support, *DEP* depressive symptoms

***p < 0.001

**Table 3:** The moderated mediation effect of stable coalition and grandparent support on the association between interparental conflict and early adolescent depressive symptoms

Variable	Equation1 (Dependent variable: SC)				Equation2 (Dependent variable: DEP)			
	*b*	*t*	*p*	*p(i)*	*b*	*t*	*p*	*p(i)*
IPC(X)	0.22	5.89	**0.001**	0.003	0.41	11.86	**0.001**	0.003
SC(M)					0.09	2.67	**0.008**	0.019
GS(U)	0.08	2.04	0.042	0.028	–0.09	–2.69	**0.007**	0.016
X*U	–0.04	–1.34	0.181	0.044	–0.12	–3.86	**0.001**	0.003
M*U					0.06	1.92	0.055	0.031
*R*^*2*^	0.06				0.28			
*F*	6.95^***^				31.07^***^			

The control variables (gender, grade and urban/rurality) were displayed in the model. *P* is the original *p*-value, *p(i)* is the critical value of significance level corrected by a B-H procedure, and the result is significant if *p* ≤ *p(i)*, significant results after B-H correction are highlighted by bold face, same below

*UCC* Unstable coercive coalition, *SC* Stable coalition, *DA* Detouring-attacking, *PC* Parentification, *DS* Detouring-supportive, *IPC* Interparental conflict, *GS* Grandparent support, *DEP* depressive symptoms

***p < 0.001

**Table 4:** The moderated mediation effect of detouring-attacking and grandparent support on the association between interparental conflict and early adolescent depressive symptoms

Variable	Equation1 (Dependent variable: DA)				Equation2 (Dependent variable: DEP)			
	*b*	*t*	*p*	*p(i)*	*b*	*t*	*p*	*p(i)*
IPC(X)	0.44	12.41	**0.001**	0.003	0.33	9.13	**0.001**	0.003
DA(M)					0.21	6.11	**0.001**	0.003
GS(U)	–0.01	–0.13	0.897	0.047	–0.08	–2.56	**0.011**	0.019
X*U	0.03	0.96	0.338	0.038	–0.11	–3.64	**0.001**	0.003
M*U					0.01	0.09	0.931	0.050
*R*^*2*^	0.20				0.28			
*F*	25.60^***^				32.46^***^			

The control variables (gender, grade and urban/rurality) were displayed in the model. *P* is the original *p*-value, *p(i)* is the critical value of significance level corrected by a B-H procedure, and the result is significant if *p* ≤ *p(i)*, significant results after B-H correction are highlighted by bold face, same below

*UCC* Unstable coercive coalition, *SC* Stable coalition, *DA* Detouring-attacking, *PC* Parentification, *DS* Detouring-supportive, *IPC* Interparental conflict, *GS* Grandparent support, *DEP* depressive symptoms

^***^p < 0.001

**Fig. 4:**
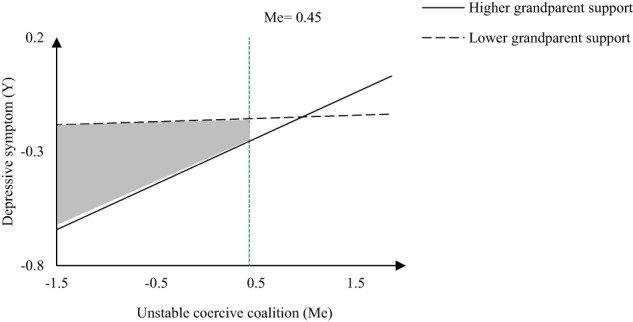
The effect of unstable coercive coalition on the depressive symptoms, moderated by grandparent support with Johnson–Neyman confidence intervals indicating significant confidence regions

A significant unstable coercive coalition × grandparent support interaction effect on depressive symptoms was also observed. Simple slope analyses indicated that adolescents with lower unstable coercive coalition reported less depressive symptoms than those with higher unstable coercive coalition when levels of grandparent support were high (*B*_*high*_ = 0.20, *SE* = 0.04, *t* = 4.58, *p* < 0.001, 95% CI = [0.12, 0.29]). However, adolescents reported relatively stable and higher depressive symptoms when levels of grandparent support were low (*B*_*low*_ = 0.02, *SE* = 0.04, *t* = 0.46, *p* = 0.65, 95% CI = [–0.07, 0.11]). The Johnson–Neyman analyses revealed that the buffering effect of grandparent support was significant when the unstable coercive coalition score was less than 0.45 (*B*_*JN*_ = –0.07, *SE* = 0.04, *t* = 1.96, *p* = 0.05, 95% CI = [–0.15, 0.00]) (region depicted by a gray background in Fig. 4).

The moderating effects of grandparent support on the associations between interparent conflict and dimensions of parent-child triangulation, as well as the interactive effects of grandparent support with stable coalition, detouring-attacking, and parentification on depressive symptoms were not found to be significant (see Tables 2–4).

### Sensitivity Analysis

To verify the stability and reliability of results, structured mediation models and moderated mediation models were rerun with interparental conflict as a latent variable, including three observed indicators: frequency, intensity, and resolution. The study found that the findings of the latent variable model were consistent with those of the observed variable model constructed in the main analyses (see Supplementary Tables 1–5 and Supplementary Figs. 1–3 in the Supplementary Material).

## Discussion

It has been unclear whether the distinct forms of parent-child triangulation might play similar mediating roles in the association between interparental conflict and adolescent depression, especially within the context of Chinese Confucianism. No prior study has further explored the potential moderating role of grandparental support in these mediating pathways. This study aimed to address these gaps. The results indicated that interparental conflict predicted adolescent depressive symptoms, and several dimensions of parent-child triangulation (unstable coercive coalition, stable coalition, and detouring-attacking) partially mediated this association. Grandparent support moderated both the direct and indirect effects of interparental conflict on early adolescent depressive symptoms.

Previous studies have shown that interparental conflict, as an essential risk factor for adolescent mental health, could positively predict adolescent depression (Park et al., 2021; Ma et al., 2020). This was also supported by the findings of this study. Therefore, interparental conflict should be considered a target in interventions aiming at preventing adolescent depressive symptoms. As for the mediating role of parent-child triangulation, the results not only supported the hypothesis of this study but also corroborated the Family Systems Theory (Minuchin, 1985), which posits that marital tension may spill over to affect parent-child relationships, and further increase the likelihood of child psychological and behavioral problems. In the present study, adolescents exposed to elevated levels of interparental conflicts tended to form an unstable or stable coalition with one parent against the other, or unite their parents by engaging in disruptive behaviors. Such triadic processes seem to defuse the tension between parents and preserve the illusory harmony of the family, but they come at the cost of placing the child at risk of depressive symptoms. This suggests that providing parents with guidance to refrain from entangling their children in marital conflicts or expressing emotional distress to them could serve as preventive measure against the onset of adolescent depression (Deng et al., 2017).

This study found that the mediating effects of parent-child triangulation on the association between interparental conflict and adolescents’ depressive symptoms vary across its distinct dimensions, among which detouring-attacking was a stronger mediator than stable and unstable coercive coalitions. This may be attributed to their different operating mechanisms (Yang, 2011). The underpinning of unstable coercive coalition and stable coalition is loyalty conflict, in which the child is compelled to choose between parents and yet conflicted about doing so (Kerig & Swanson, 2010). On the other hand, the underpinning of detouring-attacking is guilt: adolescents often bear the blame from both parents, which makes them responsible for the interparental conflict, thereby increasing self-blame and shame, a kind of subtle distress and inner turmoil (Minuchin, 1974). In both unstable and stable coalition, adolescents might still maintain a relatively positive relationship with one of their parents. In detouring-attacking, the relationships between adolescents and both parents are highly likely to be adversely affected, potentially resulting in negative experiences for the adolescents. The results of this study also revealed that higher levels of interparental conflict predicted lower levels of detouring-supportive and parentification triangulation in adolescents. Faced with high interparental conflict, family members may be emotionally alienated and preoccupied with anger, dissatisfaction, insecurity or sadness, thereby failing to take care of the family. However, such reduced levels of detouring-supportive behavior and parentification were not linked with adolescents’ depressive symptoms. Unlike other dimensions of parent-child triangulation, detouring-supportive and parentification did not mediate the association between interparental conflict and adolescents’ depressive symptoms. This highlights the value of distinguishing different aspects of parent-triangulation to advance our understanding of its potentially complex role.

Of greater importance are the mixed findings pertaining to the role of parentification. Most western studies indicated a deleterious impact of parentification on adolescents’ emotional and behavioral development, which was related to negative social adjustment (Peris et al., 2008; Williams & Francis, 2010). On the contrary, Chinese studies found that parentification improved adaptability and coping ability, and was positively related to social adjustment (Wang & Wang, 2014; Wang et al., 2017). This study further indicated that elevated parentification did not beget higher depressive symptoms. Additionally, the negative effect of interparental conflict manifested differently in Western and Chinese contexts. In Western societies, interparental conflict was related to an increase in parentification, while in the Chinese context, it was related to a decrease in parentification. As mentioned in the introduction, one reason for this discrepancy may be the cultural differences between the East and the West (Wang et al., 2014). Parentification is viewed as an age-inappropriate behavior and a kind of boundary dissolution that is problematic for child development in the West. However, influenced by the Confucian culture, parentification is seen as a positive behavior to be encouraged in China. Chinese children and adolescents tend to deem that providing parents with emotional support and maintaining family harmony is their responsibility. These findings highlight the importance of considering cultural values in future research.

In contrast to detouring-attacking, detouring-supportive was negatively associated with adolescents’ depressive symptoms, although it did not mediate the relation between interparental conflict and depressive symptoms. In detouring-attacking, children are often rejected or perceived as problematic by their parents, whereas in detouring-supportive parents typically unite to care for a child perceived as special or in need. Detouring-attacking aligns with the spillover hypothesis, positing that interparental conflicts could lead to increased negativity between parents and children. However, detouring-supportive is in line with the compensatory hypothesis, suggesting that interparental conflicts might foster relatively positive parent-child relationships (Kerig & Swanson, 2010). Given the scarcity of empirical literature on detouring-supportive in both Western and Chinese contexts, more studies are needed to investigate whether cultural differences potentially influenced its association with adolescent depressive symptoms.

Turning to the moderating role of grandparent support, this study found that grandparent support indeed played a partially protective role in adolescent adjustment. Both the direct effect and the second half pathway of interparental conflict on early adolescent depressive symptoms were moderated by grandparent support. Specifically, grandparent support alleviated the detrimental impact of an unhealthy family process (interparental conflict and unstable coercive coalition) on adolescent depressive symptoms. In agreement with the Stress-Buffering effect model (Rueger et al., 2016), social support was an essential protective factor for adolescents and could effectively assuage the adverse impact of stressful events on adolescents’ psychosocial development. The Emotional Security Theory (Davies & Cummings, 1994) also posited that the social support provided in close relationships, such as grandparent support, could help individuals regulate destructive emotions, share pressure, and provide them with advice and material assistance (Huo et al., 2018). Grandparents could provide security and love to their grandchildren and effectively attenuate the negative impact of interparental conflict and unstable coercive coalition on adolescents (Van Heerden & Wild, 2018).

However, the moderating effect of grandparental support was limited. Grandparent support could not play a moderating role when adolescents were involved in the triangulation in the form of detouring-attacking and stable coalitions. Both of these forms were more harmful to the adolescent than unstable coalitions. It might be more effective for interventions aiming to reduce interparental conflict and prevent the emergence of negative parent-child triangulation. The results also showed that grandparent support was positively associated with parentification, detouring-supportive, and unstable coercive coalition. Adolescents with higher grandparental support were more likely to engage in interparental conflicts in a less negative, and even positive, manner. Considering the common involvement of grandparents in parenting within the Chinese context, these findings imply that grandparental support might also affect adolescents’ depressive symptoms by influencing their engagement in interparental conflict, rather than solely acting as a moderating factor. Future studies could benefit from employing a longitudinal design to more comprehensively explore the multifaceted role of grandparental support in adolescent development.

This study has several strengths, including examining the different dimensions of parent-child triangulation separately and comprehensively, and assessing marital relationship, parent-child relationship, and grandparent-grandchild relationship simultaneously. By investigating the mediating role of parent-child triangulation and the moderating role of grandparent support, the findings provided a more nuanced understanding of the linkage between interparental conflict and adolescent depressive symptoms. However, several limitations of the study should be acknowledged. First, a cross-sectional design was used to explore the concurrent association between interparental conflict and depressive symptoms, which did not provide sufficient evidence for a causal relation. Further research with a longitudinal design is warranted. Second, all key measures used in this study were based on adolescents’ self-reports. Although the analysis indicated the risk of common method bias was low, and adolescents’ perceptions have been shown to be a better and stronger predictor of their well-being than parents’ perceptions (Goldner et al., 2022), future studies might benefit from including additional respondents as parent-adolescent discrepancies in perceptions of family functioning could also predict adolescents’ psychological adjustment (Fosco et al., 2021; Human et al., 2016). Third, the sample was recruited from a typical normative population and was relatively well-functioning with low levels of interparent conflict and depressive symptoms. Effects might have been even stronger if a more diverse sample was used. Fourth, the age range of the study sample was concentrated in early adolescence. The findings might differ when the sample is older or younger, and future studies can examine cohorts with broader age ranges to evaluate whether there are age-related differences in the association among the key variables of this study. Fifth, the measurement of grandparent support was based on adolescents’ subjective feelings, and did not account for the frequency of their contact with grandparents. The frequency of contact is also an important indicator for assessing grandparent support. In future research, comprehensive measurement of grandparent support incorporating the objective and subjective indicators might provide valuable insights. Consistent with existing research (Zhang et al., 2022), adolescents reported their overall grandparent support rather than specifying support from a particular grandparent in the present study. It might also be helpful to separately examine the unique contributions of paternal grandmother, paternal grandfather, maternal grandmother, and maternal grandfather to adolescent development in future studies. Finally, this study only included Chinese adolescents. Hence, this research results may not generalize to adolescents in other cultures.

## Conclusion

There exists a strong need to examine the combined influence exerted by marital relationships, parent-child interactions, and grandparent-grandchild dynamics on adolescents’ depressive symptoms. A notable ambiguity persists concerning whether distinct forms of parent-child triangulation might play a similar role in mediating the association between interparental conflict and early adolescent depressive symptoms within the context of Chinese Confucianism. This study addressed these gaps by investigating the mediating role of the five dimensions of parent-child triangulation in the association between interparental conflict and early adolescent depressive symptoms, and the moderating effect of grandparent support on these mediating pathways. The results demonstrated that adolescents exposed to greater interparental conflicts tended to establish an unstable or stable coalition with one of their parents against the other or unite their parents by engaging in disruptive behaviors. However, these strategies come at the expense of putting the child at risk of depressive symptoms. Unlike Western societies, the Chinese context revealed a unique association between interparental conflict and a decrease in parentification. Grandparent support alleviated the adverse impact of an unhealthy family dynamic (interparental conflict and unstable coercive coalition) on adolescent depressive symptoms.

### Supplementary Information

Electronic Supplementary Materials
