# Supplementary material for: Interparental Conflict and Early Adolescent Depressive Symptoms: Parent-Child Triangulation as the Mediator and Grandparent Support as the Moderator
Source: J Youth Adolesc. 2023 Dec 13;53(1):186–99. doi: 10.1007/s10964-023-01923-2 (PMC10761398; doi:10.1007/s10964-023-01923-2)
Supplement: Supplementary file 1 — Electronic Supplementary Materials [file 10964_2023_1923_MOESM1_ESM.docx]

**Supplementary Materials for Interparental Conflict and Early Adolescent Depressive Symptoms: Parent-Child Triangulation as the Mediator and Grandparent Support as the Moderator**

 Meiping Wang, Shan Sun, Xiaojie Liu, Yang Yang, Chunyu Liu, Aodi Huang, Siwei Liu

**Supplementary analysis**

To verify the stability and reliability of our results, structured mediation models and moderated mediation models were rerun with interparental conflict as a latent variable including three observed indicators: frequency, intensity, and resolution. We found that the findings of the latent variable model were consistent with those of the explicit variable model constructed in the main analyses (see Table 1 to 5 and Figure 1 to 3).

**Testing for Mediation effect (interparental conflict as a latent variable)**

We constructed a series of mediation models with parent-child triangulation as the mediating variable (see Figure 1). All models were found to fit the data well (see table 1).

UCC

0.23^***^

0.12^***^

0.45^***^

DEP

SC

0.22^***^

0.09^*^

0.48^***^

DEP

DA

0.47^***^

0.17^***^

0.42^***^

DEP

DS

–0.07

–0.02

0.49^***^

DEP

PC

–0.32^***^

–0.06

0.49^***^

DEP

**Fig. 1** Testing the mediating effect of parent-child triangulation on the association between interparental conflict and early adolescent depressive symptoms when interparental conflict is used as a latent variable. The numbers were standardized regression coefficients. For the sake of brevity, the control variables (gender, grade and urban/rurality) and the observed indicators of latent variables (frequency, intensity, and resolution) were not shown in the figure. UCC = Unstable coercive coalition; SC = Stable coalition; DA = Detouring-attacking; PC = Parentification; DS = Detouring-supportive; IPC = Interparental conflict; GS = Grandparent support; DEP = depressive symptoms.

^*^*p* < 0.05, ^***^ *p* < 0.001.

| **Table1** Mediation models fit index. | | | | | |
| --- | --- | --- | --- | --- | --- |
| **Mediator** | ***χ^2^ /df*** | **CFI** | **TLI** | **RMSEA** | **SRMR** |
| Model1: UCC | 2.22 | 0.98 | 0.97 | 0.04 | 0.03 |
| Model 2: SC | 1.85 | 0.99 | 0.98 | 0.03 | 0.03 |
| Model 3: DA | 2.02 | 0.99 | 0.98 | 0.04 | 0.03 |
| Model 4: DS | 2.07 | 0.98 | 0.98 | 0.04 | 0.03 |
| Model 5: PC | 3.00 | 0.97 | 0.96 | 0.05 | 0.04 |
| *Note.* UCC = Unstable coercive coalition; SC = Stable coalition; DA = Detouring-attacking; PC = Parentification; DS = Detouring-supportive; IPC = Interparental conflict; GS = Grandparent support; DEP = depressive symptoms. | | | | | |

**Testing for Moderated Mediation (interparental conflict as latent variable)**

We constructed three moderated mediation models with unstable coercive coalition, stable coalition, and detouring-attacking as the mediator and grandparent support as the moderator. All the models fit the data well (see table 2).

| **Table 2** Moderated mediation models fit index. | | | | | |
| --- | --- | --- | --- | --- | --- |
| **Mediator** | ***χ^2^ /df*** | **CFI** | **TLI** | **RMSEA** | **SRMR** |
| Model1: UCC | 1.76 | 0.99 | 0.98 | 0.03 | 0.03 |
| Model 2: SC | 1.91 | 0.98 | 0.98 | 0.04 | 0.04 |
| Model 3: DA | 2.00 | 0.98 | 0.98 | 0.03 | 0.04 |
| *Note.* UCC = Unstable coercive coalition; SC = Stable coalition; DA = Detouring-attacking. | | | | | |

A significant interaction between interparental conflict and grandparent support on depressive symptoms was found in each model (see Table 3-5), with a simple slope analysis as shown in Figure 2. A signiﬁcant unstable coercive coalition × grandparent support interaction effect on depressive symptoms was also observed (see Table 3), with a simple slope analysis as shown in Figure 3.

| **Table 3** The moderated mediation effect of unstable coercive coalition and grandparent support on the association between interparental conflict and early adolescent depressive symptoms. | | | | | | | | |
| --- | --- | --- | --- | --- | --- | --- | --- | --- |
| **Variable** | **Equation1 (Dependent variable: UCC)** | | | | **Equation2 (Dependent variable: DEP)** | | | |
|  | ***b*** | ***t*** | ***p*** | ***p(i)*** | ***b*** | ***t*** | ***p*** | ***p(i)*** |
| IPC(X) | 0.38 | 6.77 | **0.001** | 0.004 | 0.56 | 10.37 | **0.001** | 0.004 |
| UCC(M) |  |  |  |  | 0.10 | 2.89 | **0.004** | 0.025 |
| GS(U) | 0.16 | 4.06 | **0.001** | 0.004 | –0.07 | –2.07 | **0.038** | 0.038 |
| X*U | 0.09 | 1.91 | 0.056 | 0.042 | –0.18 | –4.14 | **0.001** | 0.004 |
| M*U |  |  |  |  | 0.09 | 3.13 | **0.002** | 0.021 |
| *Note.* The control variables (gender, grade and urban/rurality) were displayed in the model. *P* is the original *p*-value, *p(i)* is the critical value of significance level corrected by the B-H procedure, and the result is significant if *p* ≤ *p(i)*, significant results after B-H correction are highlighted by bold face, same below. UCC = Unstable coercive coalition; IPC = Interparental conflict; GS = Grandparent support. | | | | | | | | |

**Fig. 2.** The moderating effect of grandparent support **Fig. 3.** The moderating effect of grandparent support

on the association between interparental conflict and on the association between unstable coercive coalition depressive symptoms. IPC = Interparental conflict; and depressive symptoms. UCC = Unstable coercive

GS = Grandparent support; DEP = depressive symptoms. coalition; GS = Grandparent support; DEP =

depressive symptoms.

Additionally, the moderating effects of grandparent support on the associations between interparent conflict and dimensions of parent-child triangulation, as well as the interactive effects of grandparent support with stable coalition, detouring-attacking on depressive symptoms were not observed (see Table 4 to 5).

| **Table 4** The moderated mediation effect of stable coalition and grandparent support on the association between interparental conflict and early adolescent depressive symptoms. | | | | | | | | |
| --- | --- | --- | --- | --- | --- | --- | --- | --- |
| **Variable** | **Equation1 (Dependent variable: SC)** | | | | **Equation2 (Dependent variable: DEP)** | | | |
|  | ***b*** | ***t*** | ***p*** | ***p(i)*** | ***b*** | ***t*** | ***P*** | ***p(i)*** |
| IPC(X) | 0.35 | 5.57 | **0.001** | 0.004 | 0.58 | 9.96 | **0.001** | 0.004 |
| SC(M) |  |  |  |  | 0.07 | 2.00 | 0.047 | 0.033 |
| GS(U) | 0.10 | 2.73 | **0.006** | 0.025 | –0.08 | –2.23 | **0.026** | 0.029 |
| X*U | –0.06 | –1.15 | 0.252 | 0.046 | –0.18 | –3.35 | **0.001** | 0.004 |
| M*U |  |  |  |  | 0.07 | 1.78 | 0.076 | 0.041 |
| *Note.* The control variables (gender, grade and urban/rurality) were displayed in the model. *P* is the original *p*-value, *p(i)* is the critical value of significance level corrected by the B-H procedure, and the result is significant if *p* ≤ *p(i)*, significant results after B-H correction are highlighted by bold face, same below. SC = Stable coalition; IPC = Interparental conflict; GS = Grandparent support. | | | | | | | | |

| **Table 5** The moderated mediation effect of detouring-attacking and grandparent support on the association between interparental conflict and early adolescent depressive symptoms. | | | | | | | | |
| --- | --- | --- | --- | --- | --- | --- | --- | --- |
| **Variable** | **Equation1 (Dependent variable: DA)** | | | | **Equation2 (Dependent variable: DEP)** | | | |
|  | ***b*** | ***t*** | ***p*** | ***p(i)*** | ***b*** | ***t*** | ***p*** | ***p(i)*** |
| IPC(X) | 0.64 | 10.58 | **0.001** | 0.004 | 0.48 | 7.31 | **0.001** | 0.004 |
| DA(M) |  |  |  |  | 0.18 | 4.05 | **0.001** | 0.004 |
| GS(U) | 0.03 | 0.71 | 0.454 | 0.038 | –0.08 | –2.17 | **0.028** | 0.029 |
| X*U | 0.02 | 0.36 | 0.660 | 0.050 | –0.18 | –3.03 | **0.001** | 0.004 |
| M*U |  |  |  |  | 0.02 | 0.53 | 0.500 | 0.041 |
| *Note.* The control variables (gender, grade and urban/rurality) were displayed in the model. *P* is the original *p*-value, *p(i)* is the critical value of significance level corrected by the B-H procedure, and the result is significant if *p* ≤ *p(i)*, significant results after B-H correction are highlighted by bold face, same below. DA = Detouring-attacking; IPC = Interparental conflict; GS = Grandparent support. | | | | | | | | |
